# Supplementary material for: Dissecting the genetic and proteomic risk factors for delirium
Source: Nat Aging. 2025 Nov 24;6(1):235–51. doi: 10.1038/s43587-025-01018-6 (PMC12823428; doi:10.1038/s43587-025-01018-6)
Supplement: Supplementary file 1 — Supplementary Table 18. [file 43587_2025_1018_MOESM1_ESM.pdf]

nature aging

Supplementary information https://doi.org/10.1038/s43587-025-01018-6

Dissecting the genetic and proteomic risk factors for delirium

In the format provided by the authors and unedited

Table of contents:

STROBE-MR checklist pp. 2-9

STROBE-MR checklist of recommended items to address in reports of Mendelian randomization studies[1 2]

Item No.	Section	Checklist item	Page No.	Relevant text from manuscript
1	**TITLE and ABSTRACT**	Indicate Mendelian randomization (MR) as the study's design in the title and/or the abstract if that is a main purpose of the study	2	Incorporating proteomic and genetic evidence via mendelian randomisation, colocalisation and druggability analyses, we indicate putatively useful drug target proteins for delirium.
	**INTRODUCTION**			
2	**Background**	Explain the scientific background and rationale for the reported study. What is the exposure? Is a potential causal relationship between exposure and outcome plausible? Justify why MR is a helpful method to address the study question		NA
3	**Objectives**	State specific objectives clearly, including pre-specified causal hypotheses (if any). State that MR is a method that, under specific assumptions, intends to estimate causal effects	4	To achieve this […] (2) tested for plasma proteome signatures of incident delirium for up to 16 years of follow-up in UK Biobank (UKB)[33] and triangulated the results using genetically supported evidence from mendelian randomisation, colocalisation and druggability analyses; […]
	**METHODS**			
4	**Study design and data sources**	Present key elements of the study design early in the article. Consider including a table listing sources of data for all phases of the study. For each data source contributing to the analysis, describe the following:		
	a)	Setting: Describe the study design and the underlying population, if possible. Describe the setting, locations, and relevant dates, including periods of recruitment, exposure, follow-up, and data collection, when available.	21-22	The UKB is a population-based prospective study, containing a rich set of genetic and phenotypic data for approximately 500,000 participants living across the United Kingdom. Participants, aged 40 to 69 years old at recruitment between 2006 – 2010, have been linked to their annually updated electronic health records, allowing longitudinal investigation of healthcare outcomes.
	b)	Participants: Give the eligibility criteria, and the sources and methods of selection of participants. Report the sample size, and whether any power or sample size calculations were carried out prior to the main analysis	26	Plasma proteome data were available in UKB for a subset of 53,075 participants. Protein measurements of 2,923 unique plasma proteins[80] were derived from blood samples taken during randomly selected participants' initial UKB assessment visit between 2006-2010. […]

					European participants from batches 0 to 6 were extracted, as they have been reported to be highly representative of the UKB European population[80]. […]<br>Delirium incident cases were defined as the participants whose first reported delirium episode was > 1 years after baseline, that is, the date of blood sample collection at the first UKB assessment visit (Data-Field 53-0.0). Delirium data were available for up to 16 years of follow-up after baseline. The final population consisted of 32,652 European participants and 2,919 plasma proteins, including 32,111 controls and 541 delirium incident cases.
		c)	Describe measurement, quality control and selection of genetic variants	28	'pQTL analysis' section
		d)	For each exposure, outcome, and other relevant variables, describe methods of assessment and diagnostic criteria for diseases	22, 26	'Delirium phenotype' section<br>Proteins were measured using the antibody-based Olink Explore 3072 proximity extension assay. […] protein measurements with >20% missing data were removed and the remaining proteins were mean-imputed, inverse-rank normalised and standardised to ensure homogeneity across the proteins.
		e)	Provide details of ethics committee approval and participant informed consent, if relevant	31	UK Biobank has approval from the North West Multi-centre Research Ethics Committee (MREC) as a Research Tissue Bank (RTB) approval.
5	**Assumptions**		Explicitly state the three core IV assumptions for the main analysis (relevance, independence and exclusion restriction) as well assumptions for any additional or sensitivity analysis	28	Mendelian randomisation (MR) is a method used to assess potential causal influence of a modifiable exposure on an outcome, often disease risk. MR analyses are based on the use of genetic variants as instrumental variables (IV). IVs are variables associated with an exposure but not with the outcome of interest through any other pathway[90,91]. Three assumptions are required for MR to be valid: (1) IVs are significantly associated with the exposure; (2) there are no confounders of the IVs and the outcome; and (3) IVs do not affect the outcome other than through the exposure (no pleiotropy)[90].

6	**Statistical methods: main analysis**		Describe statistical methods and statistics used		
		a)	Describe how quantitative variables were handled in the analyses (i.e., scale, units, model)	26	Proteins were […] inverse-rank normalised and standardised (mean zero standard deviation 1) to ensure homogeneity across the proteins.
		b)	Describe how genetic variants were handled in the analyses and, if applicable, how their weights were selected	28-29	2,464 proteins were significantly associated with at least one genetic variant (p-value < $5x10^{-8}$). Per protein, independent pQTLs were obtained through linkage disequilibrium (LD)-based clumping, including variants with LD r^2 < 0.2 within ±250 kb windows of the top associated pQTLs. LD-clumping was done using PLINK (v1.90b7.2)[90]. […]<br><br>Genetic instruments for protein exposure traits comprised of the per-protein independent pQTLs, as derived from our pQTL analysis in UKB (p-value < $5x10^{-8}$, LD r^2 < 0.2). To further ensure no weak instrument bias in our genetic instrument pQTLs - relevant to MR assumption (1) - we also excluded pQTLs with an F-statistic < 10 in their per-protein exposure GWAS. F-statistics were approximated as $F = \frac{\widehat{\gamma_j^2}}{\sigma_j^2}$ for each pQTL j, where $\hat{\gamma}$ is the pQTL-exposure effect size and σ its respective standard error[124]. To minimise chances of including genetic instruments with pleiotropic effects, we included only (a) pQTLs associated with less than 5 proteins (p-value < $5x10^{-8}$), and (b) cis-pQTLs, that is pQTLs in close proximity with their associated protein's encoding gene. cis-pQTLs were defined as being located within ±1Mb (10^6 base pairs) of the protein-encoding gene's coding region. Protein-encoding genes' start and end positions were derived as reported in the metadata from Sun et al[110].<br><br>Genetic associations of the pQTLs with delirium were derived from our UKB-EUR GWAS excluding the individuals in the proteomic set, to avoid sample overlap in our two-sample setting ($n_{cases} = 6{,}650$; $n_{controls} = 354{,}251$). The same procedure as our full UKB-EUR GWAS was followed, as described.

		c)	Describe the MR estimator (e.g. two-stage least squares, Wald ratio) and related statistics. Detail the included covariates and, in case of two-sample MR, whether the same covariate set was used for adjustment in the two samples	22-23, 28-29	The covariates considered for the UKB GWAS included: age, sex, genotyping batch (Data-Field 22000) and the first 20 pre-computed genomic principal components (data-field 22009). Here, age was defined as age at first delirium occurrence for cases and age at last data freeze (31 October 2022) or age at death for controls. […]<br>‘pQTL analysis’ section<br>For our main MR analysis, 1989 proteins were tested in total. The inverse variance weighted (IVW) method was used[122] to assess causal estimates between 1,738 proteins with > 1 cis-pQTLs and delirium. For 251 proteins with 1 cis-pQTLs the Wald ratio method was used.
		d)	Explain how missing data were addressed	22	In UKB, the set of imputed genotypes was used[94] (Data-Field 22828), filtered to include variants with > 5 minor alleles in cases and controls, imputation score > 0.5, missingness rate < 3% and deviation from Hardy-Weinberg Equilibrium (HWE) with p-value < 10^{-6}. Individuals were filtered to include those with missingness rate < 5%, […]
		e)	If applicable, indicate how multiple testing was addressed	29	MR associations were considered significant at a false discovery rate corrected p-value (FDR q) < 0.05 in our main IVW or Wald ratio MR analysis.
7	**Assessment of assumptions**		Describe any methods or prior knowledge used to assess the assumptions or justify their validity	29	Genetic instruments for protein exposure traits comprised of the per-protein independent pQTLs, as derived from our pQTL analysis in UKB (p-value < 5×10^{-8}, LD r^2 < 0.2).<br>To further ensure no weak instrument bias in our genetic instrument pQTLs - relevant to MR assumption (1) - we also excluded pQTLs with an F-statistic < 10 in their per-protein exposure GWAS. F-statistics were approximated as $F = \frac{\widehat{\gamma_j^2}}{\sigma_j^2}$ for each pQTL j, where $\hat{\gamma}$ is the pQTL-exposure effect size and σ its respective standard error[124].<br>To minimise chances of including genetic instruments with pleiotropic effects, we included only (a) pQTLs associated with less than 5 proteins (p-value < 5×10^{-8}), and (b) cis-pQTLs, that is pQTLs in close proximity with their associated protein’s encoding gene. cis-pQTLs were defined as being located within ±1Mb (10^6

				base pairs) of the protein-encoding gene's coding region. […]<br><br>The MR-Egger intercept and the Cochran's Q tests were used to assess for the presence of horizontal pleiotropy and heterogeneity in the IVs respectively[124–126]
8	**Sensitivity analyses and additional analyses**	Describe any sensitivity analyses or additional analyses performed (e.g. comparison of effect estimates from different approaches, independent replication, bias analytic techniques, validation of instruments, simulations)	29	We further employed the weighted median (WM), maximum likelihood and MR-Egger sensitivity MR methods[123], to test for consistency of estimates with our main analysis. The MR-Egger intercept and the Cochran's Q tests were used to assess for the presence of horizontal pleiotropy and heterogeneity in the IVs respectively[124–126]. […]<br><br>For significant proteins, IVW or Wald ratio MR were repeated using delirium in the dementia-free UKB sub-cohort as outcome GWAS, again using an FDR q threshold of 0.05.<br><br>Finally, the FinnGen delirium GWAS was used as a replication MR outcome set, following the same MR framework as described. A significance threshold of p-value < 0.05 was used in the replication MR
9	**Software and pre-registration**			
	a)	Name statistical software and package(s), including version and settings used	29	All MR analyses were conducted using the TwoSampleMR R package (version 0.5.11)[95].
	b)	State whether the study protocol and details were pre-registered (as well as when and where)		NA
	**RESULTS**			
10	**Descriptive data**			
	a)	Report the numbers of individuals at each stage of included studies and reasons for exclusion. Consider use of a flow diagram	Supplementary Data 3	
	b)	Report summary statistics for phenotypic exposure(s), outcome(s), and other relevant variables (e.g. means, SDs, proportions)	Supplementary Table 1, page 26	protein measurements with >20% missing data were removed and the remaining proteins were mean-imputed, inverse-rank normalised and

					standardised (mean zero standard deviation 1) to ensure homogeneity across the proteins.
		c)	If the data sources include meta-analyses of previous studies, provide the assessments of heterogeneity across these studies		NA
		d)	For two-sample MR:<br>i. Provide justification of the similarity of the genetic variant-exposure associations between the exposure and outcome samples<br>ii. Provide information on the number of individuals who overlap between the exposure and outcome studies	29	Genetic associations of the pQTLs with delirium were derived from our UKB-EUR GWAS excluding the individuals in the proteomic set, to avoid sample overlap in our two-sample setting ($n_{cases} = 6{,}650$; $n_{controls} = 354{,}251$).
11	**Main results**				
		a)	Report the associations between genetic variant and exposure, and between genetic variant and outcome, preferably on an interpretable scale	Supplementary Data 3	
		b)	Report MR estimates of the relationship between exposure and outcome, and the measures of uncertainty from the MR analysis, on an interpretable scale, such as odds ratio or relative risk per SD difference	Figure 7,<br>Supplementary Tables 12-13	
		c)	If relevant, consider translating estimates of relative risk into absolute risk for a meaningful time period		NA
		d)	Consider plots to visualize results (e.g. forest plot, scatterplot of associations between genetic variants and outcome versus between genetic variants and exposure)	Figure 7	
12	**Assessment of assumptions**				
		a)	Report the assessment of the validity of the assumptions	Supplementary Tables 12-13	
		b)	Report any additional statistics (e.g., assessments of heterogeneity across genetic variants, such as I^2, Q statistic or E-value)	Supplementary Tables 12-13	
13	**Sensitivity analyses and additional analyses**				
		a)	Report any sensitivity analyses to assess the robustness of the main results to violations of the assumptions	Supplementary Tables 12-13	
		b)	Report results from other sensitivity analyses or additional analyses	Supplementary Tables 12-13	

		c)	Report any assessment of direction of causal relationship (e.g., bidirectional MR)		NA
		d)	When relevant, report and compare with estimates from non-MR analyses	Figure 7, Table 1	
		e)	Consider additional plots to visualize results (e.g., leave-one-out analyses)		NA
	**DISCUSSION**				
14	**Key results**		Summarize key results with reference to study objectives	20	In terms of delirium treatment, we explored whether our identified proteins could be suitable drug targets. For this, we triangulated our proteomic study findings with proteome-wide mendelian randomisation (MR) and colocalisation analyses. Incorporating genetics in drug target identification, e.g. through MR, is particularity attractive, as drugs with genetic support are more likely to be successful in clinical trials[82,83] or can offer drug repurposing opportunities[84]. […] Results should be interpreted with caution.
15	**Limitations**		Discuss limitations of the study, taking into account the validity of the IV assumptions, other sources of potential bias, and imprecision. Discuss both direction and magnitude of any potential bias and any efforts to address them	20	It should be noted that liberal p-value thresholds – up to $p < 0.05$ – were used to indicate proteomic support for our druggability assessment, and some proteins (e.g. PON3 and ADAM8) were not replicated in our replication MR, although the same effect direction was still observed (Table 1 and Supplementary Data 1). Results should be interpreted with caution.
16	**Interpretation**				
		a)	Meaning: Give a cautious overall interpretation of results in the context of their limitations and in comparison with other studies	21	Our plasma proteome analysis supports previous findings and discovers putatively novel proteins implicated to delirium, some of which suggested for therapeutic applications.
		b)	Mechanism: Discuss underlying biological mechanisms that could drive a potential causal relationship between the investigated exposure and the outcome, and whether the gene-environment equivalence assumption is reasonable. Use causal language carefully, clarifying that IV estimates may provide causal effects only under certain assumptions	19-20	Our findings have notable clinical and biological clinical implications. […] Results should be interpreted with caution.

		c) Clinical relevance: Discuss whether the results have clinical or public policy relevance, and to what extent they inform effect sizes of possible interventions	19-20	Our findings have notable clinical and biological clinical implications. […] Results should be interpreted with caution.
17	**Generalizability**	Discuss the generalizability of the study results (a) to other populations, (b) across other exposure periods/timings, and (c) across other levels of exposure		
	**OTHER INFORMATION**			
18	**Funding**	Describe sources of funding and the role of funders in the present study and, if applicable, sources of funding for the databases and original study or studies on which the present study is based	31	‘Acknowledgements’ section
19	**Data and data sharing**	Provide the data used to perform all analyses or report where and how the data can be accessed, and reference these sources in the article. Provide the statistical code needed to reproduce the results in the article, or report whether the code is publicly accessible and if so, where	31	‘Data availability’ and ‘Code availability’ sections
20	**Conflicts of Interest**	All authors should declare all potential conflicts of interest		NA

This checklist is copyrighted by the Equator Network under the Creative Commons Attribution 3.0 Unported (CC BY 3.0) license.

1. Skrivankova VW, Richmond RC, Woolf BAR, Yarmolinsky J, Davies NM, Swanson SA, et al. Strengthening the Reporting of Observational Studies in Epidemiology using Mendelian Randomization (STROBE-MR) Statement. JAMA. 2021;under review.

2. Skrivankova VW, Richmond RC, Woolf BAR, Davies NM, Swanson SA, VanderWeele TJ, et al. Strengthening the Reporting of Observational Studies in Epidemiology using Mendelian Randomisation (STROBE-MR): Explanation and Elaboration. BMJ. 2021;375:n2233.
