# Supplementary material for: Dissecting the genetic and proteomic risk factors for delirium
Source: Nat Aging. 2025 Nov 24;6(1):235–51. doi: 10.1038/s43587-025-01018-6 (PMC12823428; doi:10.1038/s43587-025-01018-6)
Supplement: Supplementary file 2 — Reporting Summary [file 43587_2025_1018_MOESM2_ESM.pdf]

nature portfolio

Corresponding author(s): Vasilis Raptis

Last updated by author(s): Sep 26, 2025

Reporting Summary

Nature Portfolio wishes to improve the reproducibility of the work that we publish. This form provides structure for consistency and transparency in reporting. For further information on Nature Portfolio policies, see our Editorial Policies and the Editorial Policy Checklist.

Statistics

For all statistical analyses, confirm that the following items are present in the figure legend, table legend, main text, or Methods section.

n/a	Confirmed	
☐	☒	The exact sample size (n) for each experimental group/condition, given as a discrete number and unit of measurement
☐	☒	A statement on whether measurements were taken from distinct samples or whether the same sample was measured repeatedly
☐	☒	The statistical test(s) used AND whether they are one- or two-sided<br>*Only common tests should be described solely by name; describe more complex techniques in the Methods section.*
☐	☒	A description of all covariates tested
☐	☒	A description of any assumptions or corrections, such as tests of normality and adjustment for multiple comparisons
☐	☒	A full description of the statistical parameters including central tendency (e.g. means) or other basic estimates (e.g. regression coefficient) AND variation (e.g. standard deviation) or associated estimates of uncertainty (e.g. confidence intervals)
☐	☒	For null hypothesis testing, the test statistic (e.g. F, t, r) with confidence intervals, effect sizes, degrees of freedom and P value noted<br>*Give P values as exact values whenever suitable.*
☐	☒	For Bayesian analysis, information on the choice of priors and Markov chain Monte Carlo settings
☒	☐	For hierarchical and complex designs, identification of the appropriate level for tests and full reporting of outcomes
☐	☒	Estimates of effect sizes (e.g. Cohen's d, Pearson's r), indicating how they were calculated

Our web collection on statistics for biologists contains articles on many of the points above.

Software and code

Policy information about availability of computer code

Data collection	No software was used for data collection.
Data analysis	All software used in the present study is publicly available. Software used for genotype pre-processing and GWAS: REGENIE (v3.2.2); LiftOver (downloaded 2023-10-22); PLINK (v1.90b4 & v2.03). For multi-trait analysis of GWAS: MTAG (v1.0.8). For meta-analysis of GWAS results: METAL (v2020-05-05). R version 4.3.0 has been used for data processing and analyses, specifically packages: glmnet (v4.1.8) for training LASSO regression models; HDL (v1.4.0) for heritability and genetic correlation analyses; TwoSampleMR (v0.5.11) for mendelian randomisation; coloc (v5.2.3) for colocalisation; medflex (v0.6-10) for mediation analysis; EnhancedVolcano (v1.18.0) for volcano plot visualisation; yardstick (v1.3.0) for (PR)AUC calculations. The GWASLab python package (version 3.4.46) was used for Manhattan plots visualisation. The KGWAS python package was used for KGWAS analysis. Custom code can be found on GitHub: https://github.com/VasiliosRaptis/deliriumGen

For manuscripts utilizing custom algorithms or software that are central to the research but not yet described in published literature, software must be made available to editors and reviewers. We strongly encourage code deposition in a community repository (e.g. GitHub). See the Nature Portfolio guidelines for submitting code & software for further information.

Data

Policy information about availability of data

All manuscripts must include a data availability statement. This statement should provide the following information, where applicable:

- Accession codes, unique identifiers, or web links for publicly available datasets
- A description of any restrictions on data availability
- For clinical datasets or third party data, please ensure that the statement adheres to our policy

Details for accessing individual-level data can be found here:
• for UK Biobank https://www.ukbiobank.ac.uk/enable-your-research/apply-for-access
• for All of Us Research Programme https://www.researchallofus.org/register

Details on obtaining delirium GWAS summary statistics used in this work can be found here:
• for FinnGen R10 release https://www.finngen.fi/en/access_results
• for MGI freeze 3 https://precisionhealth.umich.edu/our-research/michigangenomics

GWAS summary statistics generated in this work are publicly available via University of Edinburgh's Datashare service [https://doi.org/10.7488/ds/8014]. Summary statistics for the proteomic, mendelian randomisation and colocalisation analyses can be found in the Supplementary Tables file and Supplementary Data 2 and 3. Source data for all figures are provided with the paper.

Research involving human participants, their data, or biological material

Policy information about studies with human participants or human data. See also policy information about sex, gender (identity/presentation), and sexual orientation and race, ethnicity and racism.

Reporting on sex and gender	Individuals of male and female biological sex were included. Analyses were adjusted for biological sex.
Reporting on race, ethnicity, or other socially relevant groupings	We included genetically inferred ancestrally-distinct populations for the following continental groups: European, Finnish, African, Admixed American (Hispanic). Black and south Asian populations in UK Biobank were derived based on self-reported ethnic background (Field 21000).
Population characteristics	The UKB is a population-based prospective study, containing a rich set of genetic and phenotypic data for approximately 500,000 participants living across the United Kingdom. Participants, aged 40 to 69 years old at recruitment, have been linked to their annually updated electronic health records, allowing longitudinal investigation of healthcare outcomes.<br><br>As described in "Genomic data in the All of Us Research Program. Nature (2024)": The All of Us research programme includes adults 18 years and older who have the capacity to consent and currently reside in the U.S. or a U.S. territory were eligible.
Recruitment	As described in "The UK Biobank resource with deep phenotyping and genomic data. Nature (2018)": (UK Biobank) participants were selected using the NHS register, and invited to volunteer for the study. Recruitment was carried out between 2007 and 2010.<br><br>As described in "Genomic data in the All of Us Research Program. Nature (2024)": (All of Us Research Program) individuals were recruited through direct participant enrollment or recruitment at one of >340 locations at US healthcare provider organizations or federally qualified community health centers.
Ethics oversight	The UK Biobank project was approved by the National Research Ethics Service Committee North West-Haydock (REC reference: 11/NW/0382). Participants provided written informed consent to participate in the UK Biobank. An electronic signed consent was obtained from the participants. This research has been conducted using the UK Biobank Resource project 788. This work uses data provided by patients and collected by the NHS as part of their care and support.<br><br>For All of Us Research Programme informed consent for all participants is conducted in person or through an eConsent platform that includes primary consent, HIPAA Authorization for Research EHRs, and Consent for Return of Genomic Results. The protocol was reviewed by the Institutional Review Board (IRB) of the All of Us Research Program. The All of Us IRB follows the regulations and guidance of the NIH Office for Human Research Protections for all studies, ensuring that the rights and welfare of research participants are overseen and protected uniformly.

Note that full information on the approval of the study protocol must also be provided in the manuscript.

Field-specific reporting

Please select the one below that is the best fit for your research. If you are not sure, read the appropriate sections before making your selection.

☒ Life sciences ☐ Behavioural & social sciences ☐ Ecological, evolutionary & environmental sciences

For a reference copy of the document with all sections, see nature.com/documents/nr-reporting-summary-flat.pdf

Life sciences study design

All studies must disclose on these points even when the disclosure is negative.

Sample size	No statistical methods were used to pre-determine sample sizes, but our sample sizes are larger to those reported in previous similar publications for delirium, and similar to previous genetic and proteomic studies in other traits. The sample sizes were as follows:<br>• For the GWAS, sample sizes for each contributing cohort are described in Supplementary Table 1. For the overall meta-analysis n = 1,059,130 individuals, including 11,931 delirium cases.<br>• For the mediation analysis, n = 407,827 in UKB EUR sub-cohort. For the sensitivity mediation analysis n = 141,864 in the UKB EUR sub-cohort.<br>• For the MTAG, n = 846,034 individuals in the discovery set and n= 120,466 individuals in the replication MTAG.<br>• For the proteomics analysis, n = 32,652 in UKB EUR, including 541 delirium cases.
Data exclusions	Genetic variants were filtered to include variants with > 5 minor alleles in cases and controls, imputation score > 0.5, missingness rate < 3% and deviation from Hardy-Weinberg Equilibrium with p-value < 10e-6.<br>Individuals were filtered to include those with missingness rate < 5%, no mismatch between reported and genetically inferred sex (Data-Field 22001), no sex chromosome aneuploidy (Data-Field 22019), no excessive heterozygosity (Data-Field 22027) and no more than ten 3rd degree relatives (Data-Field 22021).<br>Those pre-established quality control steps were implemented to minimise the risk of biases introduced by low quality data, non-random data missingness and data labeling mistakes.
Replication	We used all available datasets in our GWAS meta-analysis to maximise statistical power, thus no replication datasets were available. For our MTAG analysis the All of Us Research Programme European ancestry dataset was used for replication of MTAG results obtained using the UKB, FinnGen and MGI datasets. For our proteomic prediction analysis, an internal random training (80%) and test (20%) set split framework was implemented in the UKB proteomic set. An additional 10-fold cross validation was implemented during the LASSO regression training step. Our mendelian randomisation analysis was replicated in the FinnGen cohort.
Randomization	Randomisation was not possible due to our study being observational. Genomic analyses were adjusted for age, sex and genomic principal components (to account for population structure). Proteomic analyses were additionally adjusted for BMI.
Blinding	Blinding was not possible due to our study being observational.

Reporting for specific materials, systems and methods

We require information from authors about some types of materials, experimental systems and methods used in many studies. Here, indicate whether each material, system or method listed is relevant to your study. If you are not sure if a list item applies to your research, read the appropriate section before selecting a response.

Materials & experimental systems

n/a	Involved in the study
☒	☐ Antibodies
☒	☐ Eukaryotic cell lines
☒	☐ Palaeontology and archaeology
☒	☐ Animals and other organisms
☒	☐ Clinical data
☒	☐ Dual use research of concern
☒	☐ Plants

Methods

n/a	Involved in the study
☒	☐ ChIP-seq
☒	☐ Flow cytometry
☒	☐ MRI-based neuroimaging

Plants

Seed stocks	NA
Novel plant genotypes	NA
Authentication	NA
